# Special Issue “Responses of Organisms to Environmental Chemical Stressors: Molecular and Cellular Insights”

**DOI:** 10.3390/ijms27010505

**Published:** 2026-01-03

**Authors:** Maria Giulia Lionetto

**Affiliations:** 1 Department of Biological and Environmental Sciences and Technologies (DiSTeBA), University of Salento, 73100 Lecce, Italy; giulia.lionetto@unisalento.it; 2 National Biodiversity Future Center (NBFC), 90133 Palermo, Italy

In recent decades, anthropogenic activities have substantially increased the release of chemical contaminants in the environment and continuous exposure to chemical stressors has become a pervasive condition for living organisms [1,2]. Chemical stressors include a broad spectrum of substances such as trace metals, pesticides, persistent organic pollutants, industrial chemicals, micro and nanoplastics, and pharmaceuticals released into the environment. These contaminants originate from agricultural runoff, industrial discharge, urban waste, and atmospheric deposition [2,3], leading to their widespread distribution in both terrestrial and aquatic environments [4,5]. To cope with these threats, living organisms activate a complex network of molecular and cellular responses such as detoxification pathways, DNA repair systems, metabolic adjustments, modulation of enzymatic activities, and changes in gene expression [6,7,8]. Investigating these responses at the molecular and cellular level provides critical insights into the mechanisms by which organisms detect, respond to, and cope with environmental contaminants, as well as the early cellular damage that can occur [9]. This knowledge is essential for identifying sensitive biomarkers of exposure, effect, and susceptibility, which can serve as early-warning tools for environmental risk assessment [10,11,12].

The Special Issue of International Journal of Molecular Science, entitled ‘Responses of Organisms to Environmental Chemical Stressors: Molecular and Cellular Insights,’ was aimed to highlight the latest research on these molecular and cellular responses, with particular attention given to innovative experimental approaches and methodologies. The contributions collected herein offer an overview of cutting-edge studies addressing diverse organisms, from invertebrates and algae to mammalian cells, and a range of stressors, including metals, microplastics, and endocrine-disrupting compounds. By integrating multi-omics, proteomics, transcriptomics, and microbiome profiling, these studies provide mechanistic insights into how organisms perceive and respond to chemical stressors, and contribute to a deeper understanding of environmental toxicology at the cellular level.

Among traditional environmental contaminants, heavy metals remain of major concern [13,14,15]. Environmental exposure to heavy metals such as cadmium is known to induce oxidative stress and disrupt key physiological processes [16,17,18,19]; however, the molecular mechanisms underlying cadmium toxicity in specialized tissues of non-model organisms are still poorly understood. In this context, Song et al. [20] performed an integrated molecular and proteomic analysis to characterize the effects of cadmium exposure on silk glands. They reported significant oxidative stress, modulation of key antioxidant enzymes, and differential expression of hundreds of proteins involved in amino acid metabolism, glutathione pathways, and redox regulation. These findings provide strong evidence that cadmium compromises physiological processes essential for silk production at multiple molecular levels and enhance understanding of heavy metal-induced stress in spiders. This work paves the way for future studies on the ecological consequences of Cd pollution.

Microplastic pollution represents an emerging and globally distributed environmental stressor [21,22,23,24,25,26]. Aquatic environments are especially exposed and vulnerable to microplastic pollution [27], whose interaction with biological systems have been widely investigated [28,29], although the molecular mechanisms and long-term effects remain incompletely understood. Yet its effects on primary producers at the cellular and molecular level are still poorly characterized. Addressing this knowledge gap, Li et al. [30] investigated how amino-modified polystyrene microplastics (PS-NH_2_) affect the diatom *Navicula* sp., revealing marked growth inhibition, chloroplast ultrastructural damage, and activation of antioxidant defenses. Transcriptomic profiling demonstrated disruptions in pathways related to porphyrin and chlorophyll metabolism, carbon fixation, glycolysis/gluconeogenesis, and endocytosis. This study provides a mechanistic link between microplastic exposure and impaired photosynthetic performance in primary producers.

The impact of microplastic exposure on host-associated microbial communities remains largely unexplored, despite their critical role in host health [31,32]. To address this issue, Auguste et al. [33] demonstrated that exposure to polyester microfibers altered the hemolymph microbiome of the mussel *Mytilus galloprovincialis*. Even short-term exposure (96 h) induced significant shifts in microbial community composition, with more pronounced changes at lower concentrations. This study highlights the sensitivity of host-associated microbiomes to textile-derived microfibers and expands the ecotoxicological paradigm to include host–microbiota interactions. The modulation of the hemolymph microbiome in *Mytilus galloprovincialis* underscores the importance of considering symbiotic microbial communities in ecotoxicology. Microbiome composition and functionality may amplify or mitigate the effects of chemical stressors on the host, suggesting a new layer of complexity in environmental risk assessment.

While many studies focus on individual contaminants, organisms in natural environments are typically exposed to complex mixtures of chemicals. The combined effects of such mixtures, particularly on reproductive cells, remain poorly understood [34,35]. The work by Mastrorocco et al. [36] examined the combined toxicity of di-(2-ethylhexyl) phthalate (DEHP) and cadmium on ovine cumulus–oocyte complexes cultured in vitro. While nuclear maturation was largely unaffected, the authors observed altered mitochondrial distribution within oocytes and increased oxidative stress in cumulus cells when both contaminants were present together, emphasizing the importance of studying environmentally relevant mixtures rather than single compounds. This study underlines the necessity of moving beyond single-compound toxicity assessments toward more realistic scenarios, where organisms are exposed to complex pollutant mixtures that may interact synergistically or antagonistically.

This Special Issue also includes comprehensive review articles that place experimental findings within broader conceptual frameworks. Khatir and Leitão [37] trace the evolution of genotoxicity studies in bivalves, from traditional cytogenetic assays to recent transcriptomic approaches. Their work underscores the central role of bivalves as sentinel species and stresses the value of multi-endpoint and multi-omics strategies in detecting early biological responses to genotoxic pollutants.

The review by Lința et al. [38] synthesizes evidence on how endocrine-disrupting chemicals (EDCs) contribute to hepatic and pancreatic toxicity. The authors emphasize mitochondrial damage, oxidative stress, and metabolic disruption as key mechanistic drivers linking EDC exposure to pathologies such as metabolic dysfunction and fatty liver disease. This review effectively bridges mechanistic toxicology with human health implications.

The six contributions in this Special Issue, although diverse in species, chemical stressors, and experimental approaches, reveal several key unifying themes that enhance our understanding of organismal responses to environmental chemical stressors. A key observation across the studies is that the integration of multi-omics and systems-level approaches emerges as a powerful strategy to unravel the complex responses of the organisms to environmental pollutants. Proteomics, transcriptomics, and microbiome profiling allow the identification of interconnected molecular pathways, reveal novel biomarkers, and provide mechanistic understanding at both cellular and subcellular levels [39,40]. Such findings highlight an additional layer of complexity that should be considered in modern ecotoxicology. The contributions also demonstrate that responses are often species- or taxon-specific, reflecting differences in physiology, metabolism, and ecological niche. This emphasizes the importance of considering a variety of organisms, including non-model species, to obtain a comprehensive understanding of environmental impacts. Moreover, the reviews highlight how molecular and cellular endpoints can bridge mechanistic toxicology with ecological and human health outcomes, linking early biochemical perturbations to broader biological consequences in a One Health framework. Collectively, these studies reinforce the concept that molecular and cellular responses can act as predictive, early-warning indicators, offering valuable tools for environmental risk assessment and informing conservation and management strategies before population-level or ecosystem-level effects occur. Overall, this Special Issue advances the field toward more integrative, realistic, and mechanistic approaches to ecotoxicology.
